# Unmatched rod contouring at the proximal end predisposes to occurrence of junctional kyphosis in early-onset scoliosis patients undergoing traditional growing rods treatment

**DOI:** 10.1186/s12891-022-05564-7

**Published:** 2022-06-29

**Authors:** Bo Yang, Liang Xu, Muyi Wang, Bin Wang, Zezhang Zhu, Yong Qiu, Xu Sun

**Affiliations:** 1 Division of Spine Surgery, Department of Orthopedic Surgery, Afliated Drum Tower Hospital, Medical School of Nanjing University, Zhongshan Road 321, Nanjing, 210008 China; 2 Medical School of Nanjing University, Nanjing, China

**Keywords:** Early-onset scoliosis, Traditional growing rods treatment, Proximal rod contouring angle, Proximal junctional kyphosis

## Abstract

**Study design:**

A retrospective case series.

**Objective:**

To investigate whether unmatched rod contouring at the proximal end predisposed to the occurrence of proximal junctional kyphosis (PJK) in early-onset scoliosis (EOS) patients after traditional growing rods (TGR) treatment.

**Summary of background data:**

TGR treatment has become a mainstay of treatment for EOS patients. PJK is one of the most common alignment-related complications.

**Methods:**

A consecutive series of EOS patients who had undergone TGR treatment were retrospectively reviewed. They were divided into PJK and non-PJK groups according to the occurrence of PJK or not. Demographic data, surgical strategies, and radiographic parameters were recorded and compared between groups. Proximal junctional angle (PJA) was defined as the angle between the caudal endplate of the UIV and the cephalad endplate of the second supradjacent vertebra above the UIV, while proximal rod contouring angle (PRCA) was defined as the angle of proximal rod contouring, which was represented by the angle between the cephalad endplate of the UIV and the caudal endplate of the second vertebra caudal to the UIV. Unmatched proximal rod contouring was regarded if the postoperative PRCA-PJA difference was greater than 5°.

**Results:**

This study finally included 73 patients. The mean age at the index surgery was 6.5 ± 2.2 years (range, 2–10 years). Mean follow-up lasted 5.0 ± 1.7 years (range, 2–9 years). They received mean 4.6 ± 1.6 lengthening procedures. There were 13 patients who were observed with PJK (18%). In comparison with the non-PJK group, the PJK group showed a larger preoperative major curve (82 ± 21° vs 70 ± 17°, *P* = 0.041) and global kyphosis (57 ± 6° vs. 44 ± 15°, *P* = 0.044). In addition, the PJK group had significantly larger postoperative PJA (10 ± 3 vs. 5 ± 3, *P*<0.001) and greater postoperative PJA-PRCA (6 ± 3 vs. 3 ± 3, *P* = 0.031). The proportion of patients with unmatched proximal rod contouring in PJK group was significantly higher than that in the non-PJK group (69% vs. 25%). Multiple logistic regression showed that preoperative GK>50°, postoperative PJA>10 and postoperative unmatched proximal rod contouring were the risk factors in predicting PJK after TGR treatment.

**Conclusion:**

Approximately 18% EOS patients experienced PJK after TGR treatment. Unmatched proximal rod contouring may be an independent risk factor of PJK occurrence, in addition to greater preoperative GK and larger postoperative PJA.

**Level of evidence:**

3.

## Introduction

Early-onset scoliosis (EOS) refers to a deformity that presents 9 years of age and younger and progressed rapidly at early stage [1]. In past decades, growing rod (GR) technique has become a mainstay of treatment for EOS patients due to its effectiveness in correcting the spinal deformity in skeletally immature pediatric population while allowing gradual growth [2]. However, patients treated with this technique need to experience repeated lengthening surgeries, which brings complications that are currently difficult to avoid, such as surgical site infection, implant-related and alignment-related complications [3–5].

Proximal junctional kyphosis (PJK) is a common alignment-related complications observed in adults and adolescents after surgery for scoliosis or kyphosis [6, 7]. Also, such a complication was observed in children after spinal deformity surgery, either with fusion or with GR. Chen [8] reported occurrence of PJK in 21 of 113 (19%) congenital scoliosis (CS) children who underwent posterior instrumented fusion. At the same time, PJK was reported in EOS patients who accepted traditional growing rods (TGR) treatment [9], with a rate ranging from 12 to 56% [9–11]. Several risk factors associated with PJK have been reported, including preoperative hyperkyphosis [12], pelvic incidence [13, 14], locations of upper instrumented vertebra (UIV) [15] and lower instrumented vertebra (LIV) [9]. Recently, a study of degenerative scoliosis in adults turned their attention to the proximal junctional area and found that the mismatch between proximal rod contour angle (PRCA) and proximal junctional angle (PJA) may be a possible risk factor of PJK [16]. A similar finding were also noted in EOS patients receiving magnetically controlled growing rods [17]. As we know, whether unmatched rod contouring at the proximal end predisposes to occurrence of PJK in patients treated with TGR has not been previously studied.

Thus, the purpose of this study was therefore to assess the incidence and risk factors of PJK, and further to determine whether unmatched proximal rod contouring is significantly related to the risk of developing PJK in postoperative patients.

## Materials and methods

### Study sample

After the approval of Institutional Review Board, a series of consecutive patients with EOS who had undergone TGR treatment between January 2009 and May 2018 were identified from our spinal deformity database. Inclusion criteria to this study were as follows: (1) patients were 9 years of age or younger at index surgery; (2) accepted dual TGR; (3) had at least 2 lengthening procedures; and (4) a minimum follow-up of 2 years with complete radiographs. Exclusion criteria were as follows: (1) previous history of spinal surgery; (2) a distal anchor at the sacrum or pelvis; (3) vertical expandable prosthetic titanium rib (VEPTR) or other growth-friendly instrumentation. Clinical demographic data including age at the index surgery, gender and etiology were recorded. Surgical data including location of UIV and LIV, anchor type (pedicle screws or hooks or hybrid), and the times and intervals of lengthening procedures were also collected. Ethical clearance was acquired from the institutional ethical committee with the reference number 2021–398-01.

### Surgical strategy

All surgeries were performed by a single surgical team who were well experienced with the TGR treatment. The surgical strategy consisted of the index surgery and the following periodical lengthening procedures. In the index surgery, UIV and LIV for each patient were determined based on evaluations of scoliotic deformity and sagittal plane of the spine. Basically, the UIV and LIV were chosen in the stable zone from T1 to T5 and L1 to L5, respectively. During the index surgery, only upper and lower ends of the instrumentation area require subperiosteally exposure. Generally, polyaxial pedicle screws were preferably used as anchors at the proximal and distal sites based on considerations on the weak bony structure in young children and the strong stress concentrated on the UIV and LIV. If the pedicle was too small, a laminar hook was placed instead. Then, dual rods were contoured to normal sagittal alignments, placed with a tandem in a submuscular manner and attached to the screws at either end. Correction of the main curve was achieved once all set screws were tightened under traction. Lengthening procedure was scheduled every 8–12 months and was performed through a small midline incision with 1-2 cm distraction. When patients enter puberty, definitive spinal fusion was considered. In some cases, the proximal and distal foundation sites were still used as end sites of fusion; however, for cases with additional junctional deformity, fusion levels should be extended properly. Somatosensory evoked potential and motor evoked potentials were applied in all surgical procedures.

### Radiographic evaluation

**Fig. 1:**
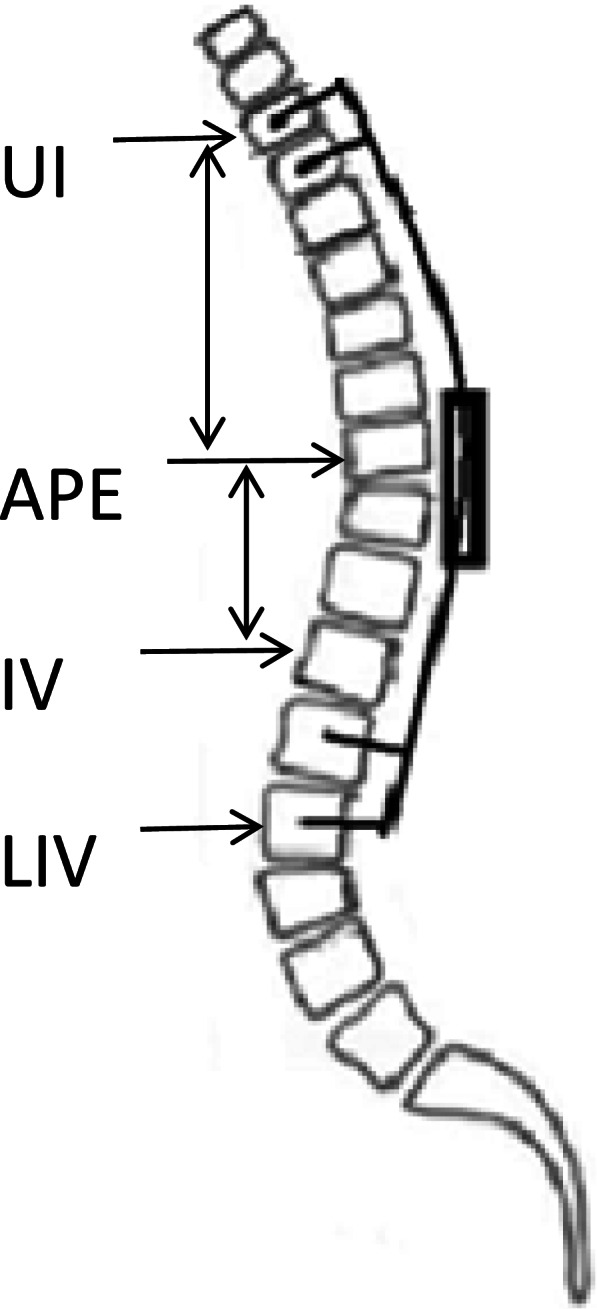
The measurement of proximal rod contouring angle (PRCA) and proximal junctional angle (PJA). PRCA: the angle between the UIV and UIV-2. PJA: the angle between the UIV and UIV + 2

All the radiographic measurements were performed on the Picture Archiving and Communications Systems (PACS) workstation. Radiographs were taken before and after the index surgery, as well as during each lengthening procedure. Standing postero-anterior radiographs were used for the measurements of the following parameters: major curve, thoracic kyphosis (TK), global kyphosis (GK), lumbar lordosis (LL), sacral slope (SS), pelvic tilt (PT), pelvic incidence (PI), sagittal vertical axis (SVA), PRCA and PJA. PJA was defined as the angle between the caudal endplate of the UIV and the cephalad endplate of the second supradjacent vertebra above the UIV; We defined the angle between the cephalad endplate of the UIV and the caudal endplate of the second vertebra caudal to the UIV as a new radiographic parameter “PRCA” (Fig. 1). And this parameter could reflect the proximal rod contouring status.

The PJA-PRCA was defined as the difference between the values of PJA and PRCA. Unmatched proximal rod contouring was regarded if the postoperative PJA-PRCA was greater than 5°.

PJK was diagnosed when the PJA met the following two criteria: (1) PJA no less than 10°; (2) at least 10° greater than preoperative measurement [18]. According to Yagi’s study [19], PJK was divided into three types: (1) Type 1, ligamentous failure; (2) Type 2, bone failure, such as fracture at or above the UIV; and (3) Type 3, implant/bone interface failure.

### Statistical analyses

Analyses were performed by SPSS version 22.0 software (SPSS Inc., Chicago, IL). Patients were divided into PJK and non-PJK group based on whether PJK developed during the follow up or not. All data were reported as mean ± standard deviation. The significance of differences between the two groups was determined by independent-sample t test. The univariate analysis by Chi-square test was used to compare the differences of categorical variables between the PJK group and non-PJK group, and further served as a screening tool to select the possible candidates for the logistic regression analysis. Subsequently, logistic regression analysis was performed on predictors with a univariate *P* value of less than 0.10 to analyze the covariate effects of the possible indicators for the development of PJK. A *P* value of less than 0.05 was considered statistically significant.

## Results

### Baseline data

A total of 73 consecutive patients (24 boys and 49 girls) were ultimately included in this study. The baseline patient characteristics are shown in Table 1. The mean age at the index surgery was 6.5 ± 2.2 years. Among them, the etiologies were classified as: congenital in 46 patients, neuromuscular in 10, idiopathic in 10, and syndromic in 7, respectively. All patients treated with TGR. Pedicle screws were placed as anchors proximally in 61 patients, while hooks in 5 and hybrid (screws in combination with hooks) in 7 respectively.

**Table 1:** Basic information

Sample size	73
Sex (M/F)	24/49
Age at index surgery (y)	6.5 ± 2.2 (2.0–10.0)
Follow up (y)	5.0 ± 1.7
Lengthening procedures	4.6 ± 1.6
Diagnosis	
Congenital	46
Idiopathic	10
Neuromuscular	10
Syndromic	7

### General results after TGR treatment

The mean lengthening interval and follow-up duration were 0.9 ± 0.1 years and 4.6 ± 1.6 years, respectively. Fourteen patients who reached skeletal maturity underwent removal of the TGR implants and definitive fusion while one patient keep close follow-up without subsequent surgery after 5 times lengthening procedures.

As shown in Table 2, the major curve experienced satisfactory correction after the index surgery (71 ± 16° VS. 33 ± 10°, *P* < 0.001) and increased slightly during subsequent lengthening surgery. The preoperative T1-S1 height increased from 24.7 ± 3.3 cm to 27.8 ± 4.1 cm after the index surgery (*P* < 0.001), and increased to 32.9 ± 4.3 cm at final follow up. On the sagittal plane, GK decreased significantly after the index surgery and increased slightly during the whole follow-up period.

**Table 2:** Comparison of radiographic parameters between preoperative, postoperative and final follow-up

	Preoperative	Postoperative	Follow up	P
Major coronal Cobb (deg.)	71 ± 16	33 ± 10	44 ± 15	0.000
Proximal junctional angle (deg.)	5 ± 3	6 ± 3	8 ± 6	0.302
T1-S1 spinal height (cm)	24 ± 3	27 ± 4	32 ± 4	0.000
T2–12 thoracic kyphosis (deg.)	40 ± 12	29 ± 10	34 ± 10	0.000
Global kyphosis (deg.)	41 ± 14	30 ± 7	40 ± 10	0.000
Lumbar lordosis (deg.)	50 ± 12	45 ± 11	46 ± 8	0.136

**Fig. 2:**
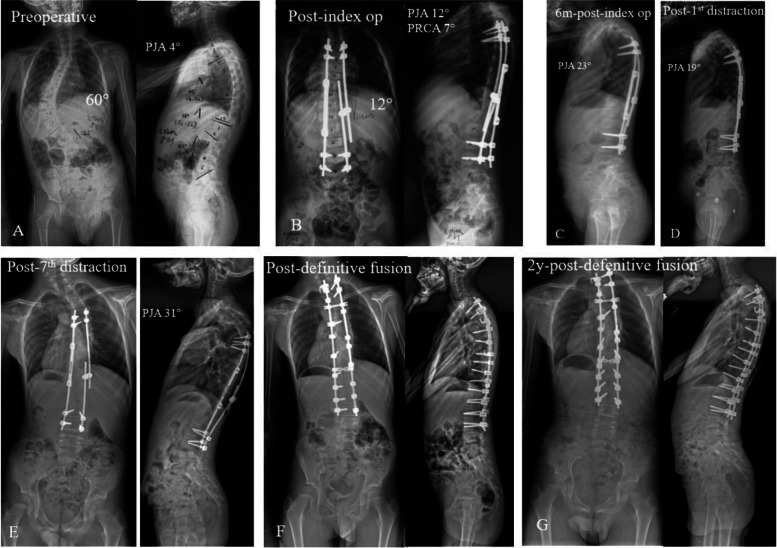
This 5-year-old boy was diagnosed with idiopathic scoliosis, showing a 60° Cobb angle and 4° PJA before surgery (**A**). After the index surgery, the radiographs showed a 12° PJA and a 7° PRCA (**B**). At 6-month follow-up, the PJA increased to 23°(**C**). After the first lengthening surgery, the PJA was 19° (**D**). After completion of 7 years of follow-up (7 lengthening), the PJA increased to 31° (**E**). This patient underwent definitive fusion at the age of 13 years old and the PJK was corrected (**F**)

Till the latest follow-up, PJK was identified in 13 patients (Fig. 2). In addition, 10 patients developed rod fracture, 2 experienced screw loosening and 1 developed distal junctional kyphosis, which were treated in the next lengthening procedures. There were 3 patients with wound superficial infections who were treated successfully by dressing changes daily and antibiotic treatment. No neurological deficit occurred in this cohort either in the index surgery or in the subsequent lengthening procedures.

### Incidence, classification, and progression of PJK

The overall incidence of PJK in our cohort was approximately 18%. There were 10 patients with type 1 PJK and 3 with type 3 PJK, respectively. None of our cohort had a type 2 PJK. Among the patients with PJK, 3 patients developed PJK within 6 months after the index surgery, 7 after the first lengthening procedures, 2 after the second lengthening procedures and 1 after the third lengthening. In the PJK group, the mean PJA increased from 6 ± 2° to 10 ± 3° after the index surgery, and continuously increased to 15 ± 2° at final follow-up. Four cases of PJK continued to progress while the other 9 cases were relatively stable. For most patients with progressive PJK, anti-kyphotic Boston brace or the Milwaukee brace was prescribed and the progress of PJK was controlled. Only one patient with progressive PJK was not effectively controlled while receiving brace treatment and was eventually corrected at definitive fusion surgery.

### Comparisons between PJK and non-PJK groups

Comparisons between PJK and non-PJK groups revealed that younger age at the index surgery was associated with the occurrence of PJK. However, no significant difference was observed in terms of gender, etiologies and number of lengthening procedures or intervals between the two groups (Table 3).

**Table 3:** Comparison in demographic and surgical data

	PJK Group	Non-PJK Group	P
Sex (male/female)	6/8	18/41	0.212
Age at index surgery (y)	5.8 ± 1.8	6.7 ± 2.2	0.028
Follow up (y)	4.8 ± 2.5	4.6 ± 1.6	0.271
Diagnosis			
Congenital	6	40	0.514
Neuromuscular	3	7	
Idiopathic	3	7	
Syndromic	2	5	
Proximal anchors (hooks/screws/hybrid)	0/11/3	5/50/4	0.113
Distraction times	4.6 ± 2.3	4.3 ± 1.2	0.121
Distraction intervals (m)	10.8 ± 2.7	11.2 ± 2.0	0.435

The radiographic characteristics of both groups are listed in Table 4. Compared with the non PJK group, the PJK group had a larger preoperative major curve (82 ± 21° VS. 70 ± 17°, *P* = 0.041), larger preoperative GK (57 ± 6° VS. 44 ± 15°, *P* = 0.044) and greater GK correction (40 ± 10% vs. 30 ± 14%, *P* = 0.035).

**Table 4:** Comparison in radiographic measurements

	PJK Group	Non-PJK group	P
PJA (°)			
Preoperative	6 ± 2	6 ± 3	0.835
Postoperative	10 ± 3	5 ± 3	<0.001
Latest follow-up	15 ± 2	6 ± 5	<0.001
PRCA (°)	3 ± 2	4 ± 3	0.781
Pre-op PJA-PRCA	3 ± 2	2 ± 2	0.801
Post-op PJA-PRCA	6 ± 3	3 ± 3	0.031
Major curve			
Preoperative	82 ± 21	70 ± 17	0.041
Postoperative	40 ± 14	39 ± 13	0.860
Latest follow-up	45 ± 15	42 ± 14	0.609
TK (°)			
Preoperative	44 ± 3	38 ± 16	0.481
Postoperative	32 ± 8	32 ± 13	0.624
Latest follow-up	35 ± 12	345 ± 29	0.677
GK (°)			
Preoperative	57 ± 6	44 ± 15	0.044
Postoperative	33 ± 8	30 ± 8	0.373
Latest follow-up	42 ± 9	37 ± 8	0.339
Correction rate (%)	40 ± 10	30 ± 14	0.035
LL (°)			
Preoperative	45 ± 16	47 ± 13	0.311
Postoperative	42 ± 16	49 ± 11	0.369
Latest follow-up	51 ± 9	52 ± 13	0.144

The change in PJA, PRCA and PJA-PRCA were also compared between the two groups. Patients in the PJK group had significantly larger PJA postoperatively (10 ± 3° VS. 5 ± 3°, *P*<0.001) and at final follow up (15 ± 2° VS. 6 ± 5°, *P*<0.001), whereas there was no difference in preoperative PJA (6 ± 2° VS. 6 ± 3°, *P* = 0.835). In addition, the postoperative PRCA was not significantly different between these two groups (3 ± 2° VS. 4 ± 3°, *P* = 0.781). With regards to the relationship between PJA and PRCA, the preoperative PJA-PRCA (3 ± 2° VS. 2 ± 2°, *P* = 0.801) showed no difference while the postoperative PJA-PRCA were significantly higher in PJK group than in non-PJK group (6 ± 3° VS. 3 ± 3°, *P* = 0.031). Also, the preoperative and postoperative values in PT, PI, LL, and SS did not differ significantly between the two groups.

### Risk factors for PJK

Univariate analysis revealed that PJK group had significantly more patients with younger age (age at index surgery≤6 yr; 62% vs. 25%), larger preoperative kyphosis (GK>50°; 62% vs. 24%), larger postoperative PJA and larger final PJA, as well as unmatched proximal rod contouring (postoperative PJA-PRCA>5°; 69% vs.25%).

As shown in Table 5, the possible candidates from the univariate analysis which were associated with PJK included age at index surgery≤6 yr, preoperative GK>50°, postoperative PJA>10° and postoperative PJA-PRCA>5°. The logistic regression analysis revealed that preoperative GK>50°, postoperative PJA>10° and postoperative PJA-PRCA>5° were independent risk factors of the occurrence of PJK (Table 6).

**Table 5:** Comparison of the risk indicators between PJK group and non-PJK group

	PJK Group	Non-PJK Group	P
Age at index surgery			
≤ 6 yr	8	15	0.011
>6 yr	5	44	
Postoperative PJA			
≤ 10	5	50	0.001
>10	8	9	
Preoperative Major curve			
≤ 80	6	37	0.270
>80	7	22	
Preoperative GK			
≤ 50	5	45	0.007
>50	8	14	
Postoperative PJA-PRCA			
≤ 5	4	44	0.002
>5	9	15

**Table 6:** Multivariate analysis of PJK risk factors

Parameters	B	SE	Wald	df	P	Exp(B)
Age at index surgery≤6 yr	0.761	0.771	0.976	1	0.323	2.141
Postoperative PJA>10	0.165	0.530	3.417	1	0.045	3.207
Postoperative PJA-PRCA>5	1.529	0.570	10.439	1	0.037	4.615
Preoperative GK>50	1.317	0.632	6.797	1	0.041	3.732

## Discussion

### Incidence and mechanism of PJK after TGR treatment

According to previous studies, the incidence of PJK in EOS patients after GR treatment varied from 12% to 56%, which was mainly due to different inclusion criteria and surgical strategies [9–11]. The incidence of PJK in our study cohort was 18% over a mean 5.0 ± 1.7 years of follow-up. It’s remarkable that PJK in this study mainly occurs after the first distraction surgery. Li [20] revealed that all patients developed PJK within the first year after VEPTR treatment. On the other hand, Wijdicks et al. [21] reported that the occurrence of PJK in patients treated with magnetically controlled growing rod increased as time progresses but had no correlation to the number of distractions.

Several studies tried to explain the patho-mechanism of PJK. A biomechanical study on adult scoliosis suggested that the dissection of posterior intervertebral elements might lead to the increase of the mechanical stresses within the junctional area. Also, the injury of the paraspinal musculature and the extensive dissected facet joints during the surgery may destroy the integrity and stability of the posterior structures of the spine [22]. According to our experience, the lengthening procedure itself is a local distraction surgery, which has a tendency to increase the kyphosis of the implant area or junctional area, eventually leading to the occurrence of PJK. In our study, the most common type of PJK was ligamentous failure which developed in 77% (10/13) patients. Our finding is in line with the study by Chen [8], but inconsistent with the study by Wang [23] who reported that implant/bone interface failure was the major type of PJK in congenital scoliosis patients receiving posterior short fusion.

### Association of unmatched rod contouring with PJK

Currently, there are still no studies reporting the mismatch between PJA and PRCA as a risk factor for PJK in EOS patients receiving TGR treatment. As a parameter to represent the proximal rod bending, the PRCA stays unchanged because the position of the screw and the rod is relatively fixed after the index surgery and allows us to indirectly measure the contouring of the proximal part of the rod. Thus, the PJA-PRCA was proposed to reflect the matching between the proximal rod contouring and supradjacent regional spinal alignment. In our study, there was no significant difference in PRCA and preoperative PJA-PRCA between the two groups. However, the PJK group has a significantly higher proportion of patients with a value of PJA-PRCA greater than 5° than that in the non-PJK group. We believe that PJK might be avoided if the postoperative PJA-PRCA matches well. But when the proximal area of the instrumentation is excessively corrected, resulting in unmatched postoperative PJA-PRCA, the risk of PJK will be greatly increased. Of note, similar findings have been observed in previous studies. Yagi et al. [19] reported that the inadequate restoration of global sagittal alignment was a significant risk factor for PJK in adult scoliosis patients undergoing long instrumented spinal fusion. Yan [16] demonstrated that mismatch between postoperative PJA and PRCA led to a proximal compensation, namely PJK in degenerative scoliosis. After reviewing 84 adolescent idiopathic scoliosis patients, Wang et al. [24] found that larger postoperative PJA-PRCA, especially those with PJA-PRCA greater than 5° were risk factors for PJK.

Consequently, we propose some possible mechanisms. A large PJA-PRCA means the proximal rod is contoured insufficiently, resulting a greater biomechanical stress concentrated in the junctional area and leading to the occurrence of PJK subsequently. Moreover, to get satisfactory correction results, the proximal region may sustain excessive forces during the surgery. According to the Hueter-Volkman principle that compression forces inhibit growth and tensile forces stimulate growth, the posterior column of spine grows faster than the anterior column after the index surgery, further aggravating the kyphosis of the proximal junctional area. Therefore, even if the PJA is less than 10° before the lengthening procedures, it may progress to more than 10° after the subsequent distraction. Another cause of PJK is related to the particularity of TGR treatment itself. TGR treatment is different from other operations in that besides the index surgery, subsequent lengthening procedures are also needed. The stress generated during the lengthening procedures will be transferred to the junctional area, making the stress concentration, and finally lead to the occurrence of PJK. This is consistent with Bess et al.^12^ findings on the complications of TGR treatment.

### General risk factors of PJK

In this study, preoperative hyperkyphosis was also identified as an independent risk factor associated with PJK. This is also consistent with previous research. Chen et al. [10] reported that hyperkyphotic EOS (TK ≥ 50°) tended to experience increased complications, such as rod fracture and PJK. Lee et al. [25] pointed out that correction surgery would impact the overall sagittal balance of the spine and PJK might be an early-stage adjustment of the overall balance compensation by the trunk. In severe kyphotic deformity, surgeons pay more attention to achieving satisfactory correction. Excessive forces were applied to the kyphosis area to the reconstruct the sagittal plane, leading to the stress concentration and eventually resulting in the occurrence of PJK.

We also found postoperative PJA>10° was a significant independent risk factor for PJK. Previously, several studies also detected larger postoperative PJA in the PJK group than the non-PJK group [9, 15]. Patients with postoperative PJA greater than 10° are more likely to develop PJK, which is helpful for early prediction and intervention. We inferred that this might be related to the improper selection of UIV or the insufficient contouring rod during the index surgery.

Although we find no significant differences between the location of UIV and LIV in PJK group and non-PJK group, some previous studies reported different opinions. Pan [15] reported UIV distal to T2 leads to an increasing stress concentration in the junctional area, which may accelerate the occurrence of PJK. Similarly, Watanabe [9] demonstrated that an LIV at or cranial to L3 may change the stress on the spine and then increase the risk of PJK.

### Clinical relevance and limitations

The clinical relevance of our study lies in emphasizing the importance of appropriate rod contouring at the proximal junctional area in preventing the occurrence of PJK. A new parameter as PRCA was defined to present the proximal rod bending in the present study. During the index surgery, the curvature of the rods should be shaped into a slightly kyphotic profile harmoniously matching the proximal spinal curve when contouring the rods. For patients with hyperkyphosis, the operation of excessive correction of kyphosis leading to the stress concentration on proximal junctional area should be avoided as much as possible. Additionally, improved level selection and attention to preservation of interspinous ligaments and suprajacent facet joints may reduce the occurrence of PJK.

Our study has several limitations. First, it was a retrospective study with a relatively short follow-up. Second, we were unable to evaluate the impact of PJK on clinical outcomes because patients are too young to fill out questionnaire by themselves. Despite these limitations, all patients were treated by the same surgical strategy and protocol which reduced the impact of the variety of surgical procedures and ensured the homogeneity of surgical results.

## Conclusion

In conclusion, the prevalence of PJK in patients with EOS undergoing traditional growing rod treatment was approximately 18%. A larger postoperative PJA-PRCA (greater than 5°) indicates relatively unmatched rod contouring at the proximal end, which can be regarded as a predictor for PJK occurrence in EOS patients. We also proved that preoperative GK>50° and postoperative PJA>10° were the risk factors for PJK.

## Data Availability

The dataset supporting the conclusions of this article is available on request – please contact the corresponding author. Administrative permission was received from the Affiliated Drum Tower Hospital of Nanjing University Medical School to access the medical records.

## Abbreviations

EOS: Early onset scoliosis
GRs: Growing rods
PJK: Proximal junctional kyphosis
PJA: Proximal junctional angle
PRCA: Proximal rod contouring angle
